# Characterization of the complete chloroplast genome of orchid family species *Paphiopedilum bellatulum*

**DOI:** 10.1080/23802359.2022.2096417

**Published:** 2022-07-18

**Authors:** Xiaobang Peng, Hang Ye, Hengzhao Liu, Zixin Zhao, Guojia Hu, Peng Zhao

**Affiliations:** a College of Urban, Rural Planning and Architectural Engineering, Shangluo University, Shangluo, China; b College of Life Sciences, Key Laboratory of Resource Biology and Biotechnology in Western China, Ministry of Education, Northwest University, Xi’an, China; c Accademia di Belle, Arti di Milano “Brera”, Milano, Italy

**Keywords:** *Paphiopedilum bellatulum*, complete chloroplast genome, phylogenetic analysis

## Abstract

The genus *Paphiopedilum* is well known as the lady’s slipper orchid in Orchidaceae family*. Paphiopedilum bellatulum* (Rchb.f.) Stein 1892, has important medicinal and ornamental value, which occurs in the tropical Asia. However, in recent decades, it was threatened with extinction by significantly reduced small population size. In this study, we sequenced and characterized the complete chloroplast genome of *P. bellatulum* based on the Illumina Hiseq platform. The size of *P. bellatulum* chloroplast genome was 156,567 bp, including a large single-copy (LSC) region of 88,243 bp, a small single-copy (SSC) region of 3652 bp, and two inverted repeat regions (IRs) of 32,336 bp. The overall GC contents of the chloroplast genome were 35.71%. A total of 122 genes were annotated, including 76 protein-coding genes, 38 transfer RNAs (tRNAs), and eight ribosomal RNAs (rRNAs). The phylogenetic analysis indicated that *P. bellatulum* formed a close relationship with another *Paphiopedilum* species *P. wenshanense*. The results will provide helpful genetic resource for further phylogenetic studies of the genus *Paphiopedilum*.

The genus *Paphiopedilum* is well known as the lady’s slipper orchid with about 96 species in the Orchidaceae family*. Paphiopedilum bellatulum* (Rchb.f.) Stein 1892, mainly occurs in Myanmar, Thailand, and southwest China as a disjunctive distribution with a small population size. *P. bellatulum* is becoming an endangered species with a rapidly reduced population size in wild habitat, and is listed in the IUCN Red List of Endangered Species (Thammasiri 2016). It is widely cultivated as horticulture plant for international trade owing to its ornamental value (Zeng et al. 2010). Although lots of valuable information and economic benefits of *P. bellatulum* have been explored through ornamental, medical, and endangered conservation studies (Ye et al. 2021), genetic study has rarely been reported for this important orchid plant (Thammasiri 2016). Therefore, in this study, we first reported the complete chloroplast genomes of *P. bellatulum* based on Illumina pair-end sequencing data. The results would provide beneficial knowledge for the utilization and conservation of *P. bellatulum* germplasm in the future, as well as supply helpful information on future genetic studies of *P. bellatulum.*

In this study, the plant sample was collected in Pu'er City, Yunnan Province (100°42′E, 23°29′N) and the voucher specimen (accession no. SK2017200) was kept in the herbarium of Northwest University (108°55′E, 34°15′N, College of Life Sciences, Hang Ye, SXSDyehang@hotmail.com). The genomic DNA was extracted from leaves using plant genomic DNA kit (Tiangen Biotech, Beijing, China). The libraries of insert sizes of ∼350 bp were constructed from randomly fragmented genomic DNA, and 150 bp paired-end reads were produced on the Illumina Hiseq platform (Illumina, San Diego, CA). After trimming, the whole chloroplast sequence of *P. bellatulum* was assembled using MITObim 1.7 program (Hahn et al. 2013) based on the *P. purpuratum* reference genome (GenBank accession number: MN535015) (Chen et al. 2019). The annotations of *P. bellatulum* chloroplast genome were performed using the online program CPGAVAS2 (Shi et al. 2019) followed by manual correction. The complete chloroplast genome sequence of *P. bellatulum* was submitted to GenBank with the accession number MN315107.

The complete chloroplast genome of *P. bellatulum* was 156,567 bp in length, which contains a pair of inverted repeat regions (IRs) with the length of 32,336 bp, a large single-copy (LSC) region with the length of 88,243 bp, and a small single-copy (SSC) region with the length of 3652 bp. The total GC content was 35.71%, while the corresponding values of the IRs, LSC, and SSC region were 39.42%, 33.26%, and 28.83%, respectively. A total of 122 genes were predicted, including 76 protein-coding genes (PCGs), 38 transfer RNA (tRNAs), and eight ribosomal RNA (rRNAs), respectively. Among these annotated genes, all rRNAs, eight PCGs (*ycf1*, *ycf2*, *rps7*, *rps15*, *rps19*, *rpl2*, *rpl23*, and *psaC*) and eight tRNAs (*trnH-GUG*, *trnR-ACG*, *trnA-UGC*, *trnI-GAU*, *trnV-GAC*, *trnL-CAA*, *trnN-GUU*, and *trnI-CAU*) were with double copies. The results of annotation showed that the majority (102 genes, 83.61%) genes contain no introns, whereas 20 genes (16.39%) contain introns.

To further study on the whole chloroplast genome of *P. bellatulum*, a total of 18 chloroplast genome sequences (16 Orchidaceae species and two Liliaceae species as outgroup were downloaded from NCBI) were used to construct the maximum-likelihood (ML) phylogenetic analysis (Figure 1). The nucleotide sequence of all chloroplast genomes had been aligned using MAFFT 7.0.0 (Katoh and Standley 2013), and the ML phylogenetic tree was conducted using the software IQ-TREE (Nguyen et al. 2015) under the best nucleotide substitution model GTR + F+I + G4 with 1000 times ultrafast bootstrap replicates. The phylogenetic analysis indicated that *P. bellatulum* was clustered into the same clade with seven other *Paphiopedilum* species, and it was closely related to *P. wenshanense* (Figure 1). These results would provide a valuable genetic foundation for the further investigation of phylogeny, evolution, and genetic studies of the important valuable species in genus *Paphiopedilum* and Orchidaceae family.

**Figure 1.**
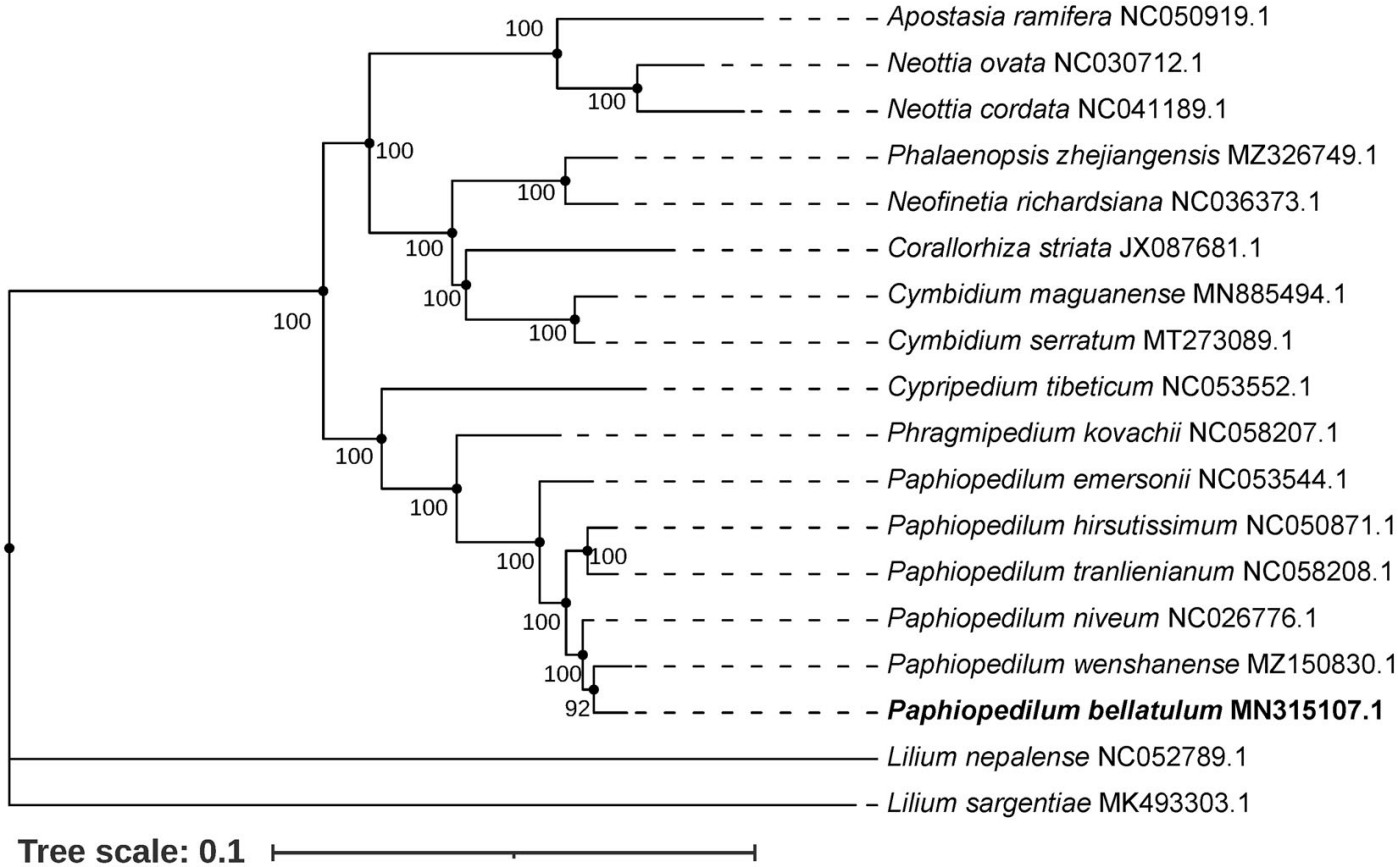
Maximum-likelihood (ML) phylogenetic tree based on 18 complete chloroplast genome sequences. The GenBank accession numbers are shown in the figure. Numbers on nodes indicated the bootstrap support values.

## Data Availability

The genome sequence data that support the findings of this study are openly available in GenBank of NCBI at https://www.ncbi.nlm.nih.gov/nuccore/MN315107 under the accession no. MN315107. The associated BioProject, SRA, and Bio-Sample numbers are PRJNA791767, SRR17318926, and SAMN24344704, respectively.
